# Complete resolution of a calcifying cystic odontogenic tumor with physiological eruption of a dislocated permanent tooth after marsupialization in a child with a mixed dentition: a case report

**DOI:** 10.1186/s12957-015-0697-0

**Published:** 2015-09-17

**Authors:** Keiji Masuda, Shintaro Kawano, Haruyoshi Yamaza, Taiki Sakamoto, Tamotsu Kiyoshima, Seiji Nakamura, Kazuaki Nonaka

**Affiliations:** Department of Pediatric Dentistry and Special Needs Dentistry, Kyushu University Hospital, 3-1-1 Maidashi, Higashi-ku, Fukuoka, 812-8582 Japan; Section of Oral and Maxillofacial Oncology, Division of Maxillofacial Diagnostic and Surgical Sciences, Faculty of Dental Science, Kyushu University, Fukuoka, Japan; Section of Pediatric Dentistry, Division of Oral Health, Growth and Development, Faculty of Dental Science, Kyushu University, Fukuoka, Japan; Laboratory of Oral Pathology, Division of Maxillofacial Diagnostic and Surgical Sciences, Faculty of Dental Science, Kyushu University, Fukuoka, Japan

**Keywords:** Calcifying cystic odontogenic tumor, Marsupialization, Pediatric dentistry

## Abstract

Here, we report the complete resolution of a calcifying cystic odontogenic tumor (CCOT) in the right mandible after marsupialization in an 8-year-old girl with a mixed dentition. Clinical, radiographic, and histopathological findings showed a simple cystic variant of CCOT in the region of the deciduous second molar, with dislocation of the permanent second premolar tooth germ. Initial treatment involved marsupialization, including extraction of the involved deciduous tooth, incision of pathological tissue, and creation of a window in the extraction socket. The crown of the dislocated second premolar was exposed at the base of the cystic cavity after marsupialization. One year and nine months later, complete bone healing and spontaneous eruption of the second premolar were observed, providing evidence of the bone regeneration capacity and tooth germ eruption potential in children. No recurrence was observed after 7 years. The findings from this case suggest that marsupialization can be successfully applied for the treatment of CCOT in children with a mixed dentition.

## Background

The 2005 World Health Organization (WHO) Classification of Odontogenic Tumors renamed calcifying odontogenic cysts (COCs) as calcifying cystic odontogenic tumors (CCOTs) [1–3]. The clinicopathological features of ghost cell odontogenic tumors (GCOTs), including CCOTs, dentinogenic ghost cell tumors (DGCTs), and ghost cell odontogenic carcinomas (GCOCs), were also defined. CCOTs can be further classified into the following four variants: simple cystic, odontoma-associated, ameloblastomatous proliferating, and CCOT associated with a benign odontogenic tumor other than odontoma [3].

CCOTs are generally benign, and the recommended treatment involves simple enucleation and curettage, regardless of the variant [2, 3]. However, enucleation and curettage may damage adjacent anatomical structures. Therefore, more conservative treatments such as marsupialization or decompression should be considered before enucleation and curettage, particularly in children. A few reports have demonstrated successful healing after marsupialization or decompression of CCOTs [4, 5].

Here we present the second case, to the best of our knowledge, of CCOT in an 8-year-old girl with a mixed dentition that was successfully treated by marsupialization only, which resulted in excellent bone healing and facilitated physiological eruption of a dislocated permanent tooth.

## Case presentation

**Fig. 1:**
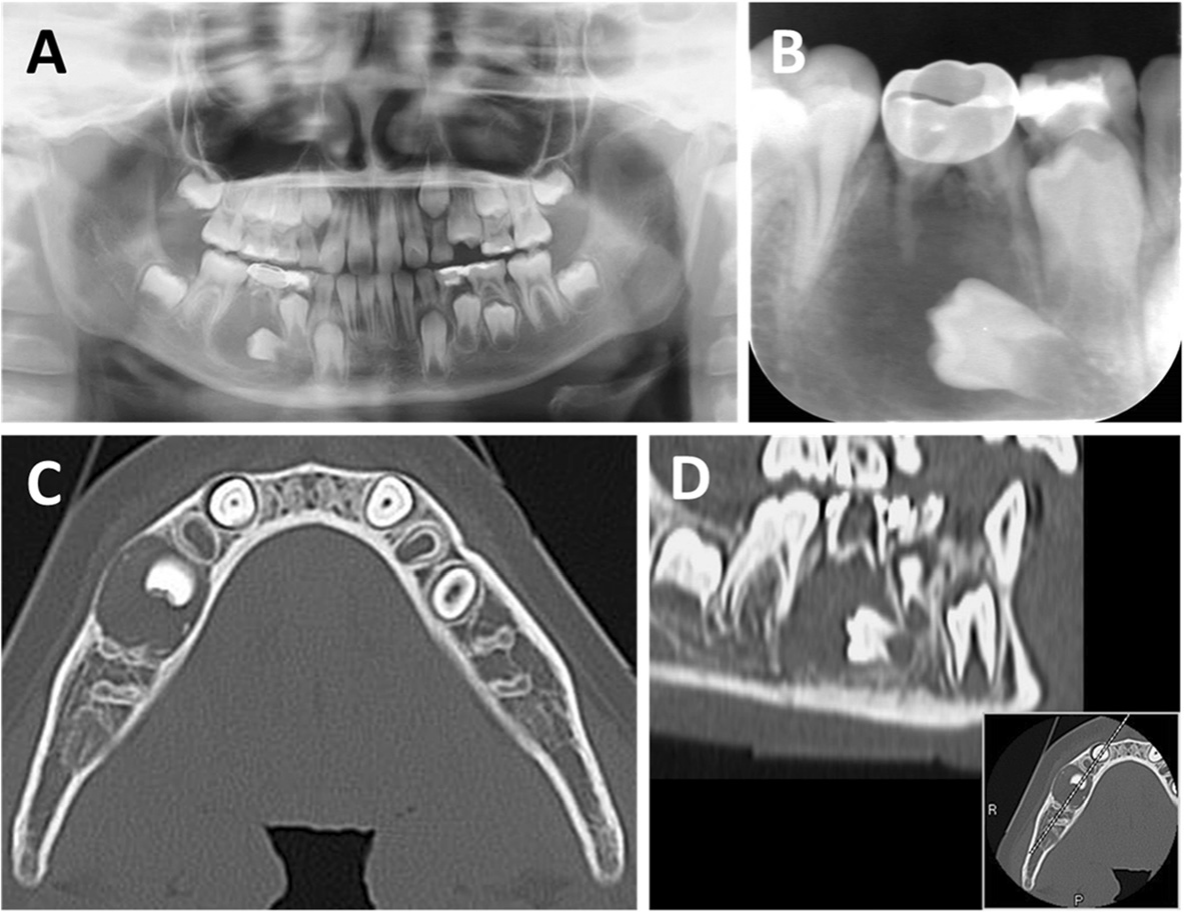
Preoperative radiographs. **a** Panoramic radiograph showing a large unilocular radiolucent lesion with a dislocated permanent premolar tooth germ in the right mandible. **b** Periapical radiograph showing partially absorbed roots and infection in the involved deciduous second molar. **c** Horizontal computed tomography view of the lesion showing small, high-density spots within a well-defined radiolucent region, with buccal and lingual cortical expansion. **d** Sagittal computed tomography view

An 8-year-old healthy girl was referred to our hospital by her general dentist who suspected the presence of an odontogenic cystic lesion in the region of the deciduous mandibular right second molar. Intraoral examination during her first visit revealed buccal bone expansion in the mandibular right molar region. Panoramic and periapical radiographs revealed a unilocular cystic radiolucency around the roots of the deciduous second molar (Fig. 1a, b). In addition, the tooth germ of the second premolar was involved and medially displaced. Computed tomography showed small high-density spots within a well-defined radiolucency, with buccal and lingual bone expansion (Fig. 1c, d). No cortical bone distraction was observed. These findings strongly suggested CCOT, although a dentigerous cyst was also a possibility.

For further confirmation of the diagnosis, incisional biopsy was performed as follows. First, needle aspiration was performed, but only a small amount of mucoid liquid was obtained, which was not adequate for cytological or microbiological examination. Then, the deciduous second molar was extracted, followed by tissue incision through the extracted socket. The crown of the developing second premolar was exposed at the base of the cystic lesion, suggesting the presence of a unicystic lesion. Finally, we created a window in the extracted socket for marsupialization to decrease the lesion size.

**Fig. 2:**
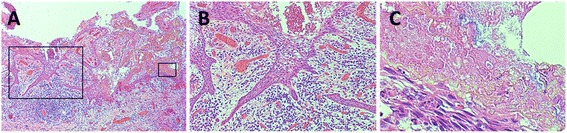
Histopathological appearance of a biopsy specimen (hematoxylin and eosin staining). **a** Low magnification, ×40. **b** Ameloblastoma-like epithelium can be observed at a higher magnification (×100) of **a**. **c** Ghost cells in various stages of maturation and foci of calcification can be observed at a higher magnification (×400) of **a**

A biopsy specimen showed inflamed granulation tissue intermingled with ameloblastoma-like epithelium (Fig. 2a, b), which contained keratin material, calcified material, and calcifying ghost cells (Fig. 2a, c). These clinical, radiographic, and histopathological findings confirmed CCOT (the simple cystic variant) accompanied by dislocation of the permanent second premolar tooth germ. We planned to perform enucleation and curettage after cyst shrinkage by marsupialization.

**Fig. 3:**
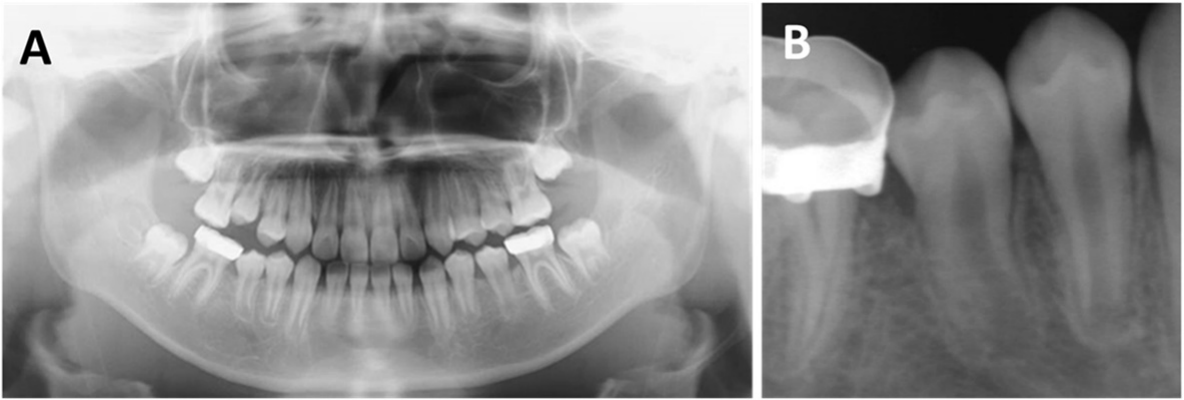
Radiographs obtained 1 year and 9 months after marsupialization. **a** Panoramic radiograph showing disappearance of the radiolucent lesion and normal eruption of the second premolar. **b** Periapical radiograph showing normal root formation in the second premolar

On postoperative day 10, the packing gauze was removed and an acrylic obturator positioned to keep the cavity open. The patient and her parents were instructed to irrigate or rinse the cavity with saline solution twice a day. After 4 months of follow-up, the size of the lesion had significantly decreased, with spontaneous eruption of the permanent second premolar. The obturator was then replaced by a lingual arch as a space maintainer, which guided the premolar into its correct position. One year and nine months later, progressive bone healing and normal eruption of the second premolar were observed on panoramic and periapical radiographs (Fig. 3); this precluded the requirement for a second surgery. No recurrence was observed during a 7-year follow-up.

## Conclusions

We reported a case involving an 8-year-old girl with the simple variant of CCOT that was successfully managed using marsupialization alone. The noninvasive features of these lesions warrant enucleation and curettage rather than extensive surgical treatment, which is often required for more aggressive neoplastic lesions such as DGCTs and GCOCs [1–3]. However, in young children, enucleation and curettage may damage the developing permanent tooth germs and important anatomical structures located adjacent to the lesion. To minimize this risk, marsupialization or decompression should be considered as the first choice of treatment before enucleation and curettage in children [6]. This conservative treatment has been shown to be highly effective for other odontogenic cystic lesions of the jaw, such as dentigerous cysts, radicular cysts, keratocystic odonotogenic tumors (KCOTs), and unicystic ameloblastomas [7–9]. Although secondary surgery such as enucleation is required when cyst shrinkage is incomplete, marsupialization or decompression may result in complete resolution of these types of cystic lesions [8, 9].

Marsupialization or decompression has been applied in only four pediatric cases of CCOTs thus far [4, 5]. All four patients had unicystic lesions accompanied by impacted teeth or odontoma-like tooth structures. However, only one case was completely resolved by a single surgical procedure involving marsupialization and removal of the calcified mass [4]; the remaining three required secondary surgery for the removal of residual tissue or odontomas [4, 5]. The present case exhibited a unicystic lesion in the region of the deciduous mandibular right second molar, with dislocation of the permanent premolar tooth germ. Odontoma-like structures were not identified, although the pulp of the deciduous second molar was necrotic, possibly because of dental caries. Therefore, our diagnosis was the simple cystic variant of CCOT that radiographically resembled an inflammatory dentigerous cyst, which is more frequently observed in patients with a mixed dentition.

The surgical procedure of marsupialization in our patient involved extraction of the involved deciduous tooth, incision of pathological tissue for microscopic examination, and creation of a window in the extracted socket. The same technique is widely used for inflammatory dentigerous cysts associated with an infected deciduous tooth and an impacted permanent successor in children with a primary or mixed dentition [9], and it may lead to complete ossification of bony defects along with successful eruption of the permanent successor in the dentigerous cyst. Therefore, relieving the pressure within the bony cyst may dramatically promote the active growth potential of bone and the eruption of the tooth germ in children.

Similar to the results for dentigerous cysts, complete bone healing and normal eruption of the dislocated permanent tooth were observed after marsupialization in our patient. Thus, in addition to an excellent capacity for bone regeneration, the involved permanent tooth germ exhibited an excellent eruptive potential because of its immature roots. Moreover, there were no odontoma-like structures or large calcified masses that could have prevented shrinkage of the lesion. Collectively, these factors predicted successful healing after the single surgical procedure of marsupialization. Although the residual mucosa was not subjected to histopathological examination, our clinical and radiographic findings suggested that the pathological cells of CCOTs disappear by undergoing metaplasic transformation to normal mucosal cells; this has also been confirmed after marsupialization or decompression of KCOTs [8, 10].

No signs of recurrence were observed during 7 years of follow-up in our patient. However, further follow-up is necessary because CCOT recurrence has been reported to occur after 1–8 years [2].

In conclusion, we reported a pediatric case of CCOT that completely resolved with subsequent physiological eruption of the involved permanent tooth by marsupialization only, illustrating the excellent potential of bone regeneration in children. Marsupialization should be considered the first choice of treatment for pediatric CCOTs in order to minimize the damage to adjacent anatomical structures caused by enucleation and curettage.

## Consent

Written informed consent was obtained from the parents of the patient for publication of this Case Report and any accompanying images. A copy of the written consent form is available for review by the Editor-in-Chief of this journal.

## Abbreviations

CCOT: calcifying cystic odontogenic tumor
WHO: World Health Organization
COCs: calcifying odontogenic cysts
GCOTs: ghost cell odontogenic tumors
DGCTs: dentinogenic ghost cell tumors
GCOCs: ghost cell odontogenic carcinomas
KCOTs: keratocystic odontogenic tumors
